# An improved method for constructing tissue microarrays from prostate needle biopsy specimens

**DOI:** 10.1136/jcp.2009.065201

**Published:** 2009-07-20

**Authors:** F McCarthy, A Fletcher, N Dennis, C Cummings, H O’Donnell, J Clark, P Flohr, R Vergis, S Jhavar, C Parker, C S Cooper

**Affiliations:** 1 Institute of Cancer Research, Male Urological Cancer Research Centre, Sutton, Surrey, UK; 2 The Royal Marsden NHS Foundation Trust, Sutton, Surrey, UK

## Abstract

**Background:**

Prostate cancer diagnosis is routinely made by the histopathological examination of formalin fixed needle biopsy specimens. Frequently this is the only cancer tissue available from the patient for the analysis of diagnostic and prognostic biomarkers. There is, therefore, an urgent need for methods that allow the high-throughput analysis of these biopsy samples using immunohistochemical (IHC) markers and fluorescence in situ hybridisation (FISH) analysis based markers.

**Methods:**

A method that allows the construction of tissue microarrays (TMAs) from diagnostic prostate needle biopsy cores has previously been reported. However, the technique only allows the production of low-density biopsy TMAs with a maximum of 20 cores per TMA. Here two methods are presented that allow the rapid and uniform production of biopsy TMAs containing between 54 and 72 biopsy cores. IHC and FISH techniques were used to detect biomarker status.

**Results:**

Biopsy TMAs were constructed from prostate needle biopsy specimens taken from 102 patients entered into an active surveillance trial and 201 patients in a radiotherapy trial. The detection rate for cancer in slices of these biopsy TMAs was 66% and 79% respectively. Slices of a biopsy TMA prepared from biopsies from active surveillance patients were used to detect multiple IHC markers and to score *TMPRSS2-ERG* fusion status in a FISH-based assay.

**Conclusions:**

The construction of biopsy TMAs provides an effective method for the multiplex analysis of IHC and FISH markers and for their assessment as prognostic biomarkers in the context of clinical trials.

Prostate cancer is the commonest cancer diagnosed in men in Western societies with over 200 000 cases diagnosed each year in the USA alone. Unfortunately established prognostic factors (Gleason score, T stage, blood PSA, cancer volume) are inadequate for predicting the precise clinical behaviour of individual patients, and there is an urgent need for new biomarker discovery. In order to test new biomarkers needle biopsy specimens taken at the time of diagnosis have been used in molecular profiling of cancer specimens, with Khor *et al*,^1^ ^2^ for example, using sections of the entire length of the formalin biopsy specimen to examine prognostic markers in patients with prostate cancer. Singh *et al*^3^ showed that the conventional tissue microarray (TMA) construction techniques described by Kononen *et al*^4^ can be used to punch out regions of the needle biopsy specimen that contained cancer and create high density arrays. However, both of these techniques are limited in that the number of slices that can be taken for biomarker analysis is limited by the width of the biopsy. In an alternative approach^5^^–^7 we have re-orientated the biopsies into a vertical orientation allowing construction of biopsy TMAs and the analysis of the markers in cross sections taken from each biopsy. An alternative method of biopsy TMA production has also been described by Datta *et al*,^8^ in which the biopsy is cut off the surface of the paraffin block, held with forceps, and placed in a hole that has been bored in a recipient wax block. A limitation of our original technique^5^ is that a maximum of 20 biopsy specimens could be included within each TMA. A particular problem is that the cutting out of small regular “checkers” of wax containing a segment of the biopsy on one face, which is required for the construction of the biopsy TMA, is technically demanding and time consuming.^5^ Here we present a simplified technique for cutting wax checkers, and describe methods for constructing biopsy TMAs containing 54–72 checkers. The success rates for constructing biopsy TMAs from patients undergoing radiotherapy and entered into active surveillance trials are reported.

## MATERIALS AND METHODS

### Clinical samples

Men with untreated, localised (clinical stage T1/2a, Gleason score ⩽3+4; PSA <15; ⩽50% positive cores) prostate cancer were managed in a prospective study of active surveillance at the Royal Marsden Hospital NHS Foundation Trust. The methods used for selecting samples for biopsy TMA construction from prostate cancer patients entered into active surveillance and from the MRC RT01 radiotherapy trial have been described previously.^6^ ^7^ All patients gave their written consent to take part in the active surveillance and radiotherapy studies, which were approved by the local research ethics committee.

### Construction of biopsy tissue microarrays

Two methods were used to construct biopsy TMAs. The two methods differ primarily in the shape and construct of the template used to accommodate the checkers and the method of embedding. The first method utilised a rubber mould that contained 54 4 mm deep, 2 mm×2 mm square rubber pegs (figure 3B, supplementary fig 1). The pegs are spaced 1 mm apart. The rubber mould was placed in a disposable wax mould (E10.6/2001/33, RA Lamb, UK) and an embedding cassette (E10, RA Lamb, UK) was positioned over the top (supplementary fig 1A–C). Paraffin wax (W1, RA Lamb, UK) at 60°C was distributed in the wax mould while the cassette was held in position by forceps (supplementary fig 1D,E). The wax mould was then allowed to solidify initially on an ice block and then in a freezer at −20°C (supplementary fig 1F,G). The resulting wax template construct accommodates 54 separate checkers (fig 3A,C). In the second method, the rubber mould contained three larger 4 mm deep pegs of 10 mm×20 mm, 8 mm×20 mm and 6 mm×20 mm (fig 3F). Paraffin wax at 60°C was distributed with a tissue embedding station (Microm EC350, Thermo-Fisher, UK) into an aluminium mould base (supplementary fig 2A) and the rubber mould was then pressed firmly into the wax and placed on a cold plate (−15°C) (Microm EC350-2, Thermo-Fisher, UK) and allowed to solidify (fig 3E, supplementary figure 2B,C). Aluminium mould base and rubber mould inserts were constructed by the Institute of Cancer Research workshops. The rubber mould inserts were made from RTV (room temperature vulcanising) rubber (EMA Model Supplies, UK). Wax checkers made as described in the text were inserted into the wax templates as shown in supplementary fig 3. The methods for binding the wax of the checkers and templates are described in supplementary fig 3.

**Figure 1:**
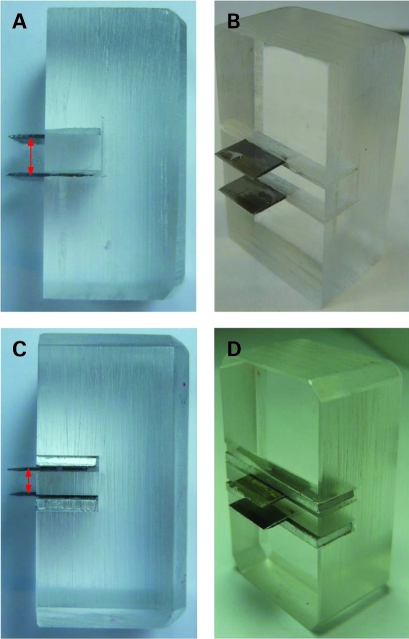
Knives used for cutting biopsy checkers. The knives each consist of parallel surgical blades fixed in translucent acrylic plastic. The figure shows knives with the blades 4 mm apart (A, B) and 2 mm apart (C, D).

**Figure 2:**
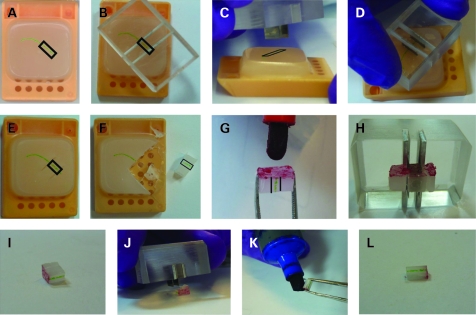
Construction of biopsy checkers. For clarity the formalin fixed prostate cancer needle biopsy has been coloured green. The position of the 4 mm length of biopsy on the surface of the wax block selected for checker construction is marked (A). The knife containing parallel blades 4 mm apart (fig 1A, B) is positioned over the wax block (B, C) so that a 4 mm length of biopsy is cut (D, E). A scalpel blade is used to cut the section of wax containing the attached 4 mm biopsy segment from the block (F). For orientation the side of the checker is marked with red ink (G). The knife containing blades 2 mm apart (fig 1C, D) is then used to make two further cuts (H–J) and for orientation an additional face of the checker is marked with blue ink (K). The final 4 mm×2 mm×2 mm checker is shown (L). The marking of faces of the checker with red and blue ink is important because it is often difficult to visualise the biopsy specimen as the checker is being cut. The entire process of checker construction takes approximately 5 minutes.

**Figure 3:**
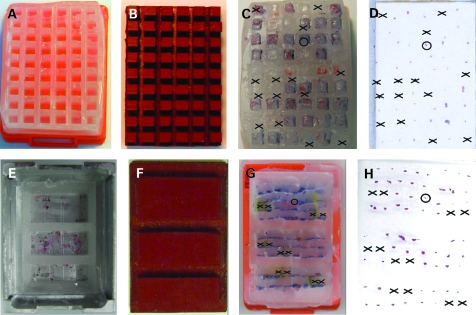
Construction of wax templates. Wax templates were constructed to accommodate 4 mm×2 mm×2 mm wax checkers. Two formats were used. In the first format (A–D) individual wells were created into which a single checker could be placed. In the second format (E–H) three larger wells (6 mm×20 mm, 8 mm×20 mm and 10 mm×20 mm) were created which together accommodated 72 checkers. The rubber moulds used to construct each template are shown respectively in (B) and (F). The detailed methods for constructing the wax templates are shown in supplementary figs 1 and 2. The methods used to insert individual checkers into the templates are shown in supplementary fig 3. (C, G) Completed biopsy tissue microarrays. (D, H) H&E stained sections of each biopsy tissue microarray (TMA). A black cross and black circle denote the position of a blank checker and of a representative biopsy core, respectively. The construction of the entire biopsy TMA from formalin fixed biopsy specimens takes 2 days.

### Immunohistochemical and FISH analysis

Sectioning and preparation of biopsy TMA blocks has been described previously.^5^ Fluorescence in situ hybridisation (FISH) detection of *ERG* gene status was carried out exactly as described previously.^9^ ^10^ Immunohistochemical analysis for H&E, p63/AMACR, Ki-67 and Hif1-α staining were performed exactly as described previously.^6^ ^11^

## RESULTS

### An improved method for the preparation of individual blocks or “checkers”

The starting point for construction of biopsy TMAs is a paraffin block containing one or more formalin fixed prostate needle biopsies. For each biopsy block the original H&E stained section is examined to confirm the diagnosis of prostate cancer and to identify the location of the cancer within the biopsy. In the original method^5^ small checkers (approx 4 mm×2 mm×2 mm) containing a 4 mm length of the biopsy specimen were roughly cut from the block with a scalpel blade. To improve the uniformity of the size of the checker and the speed of production we have devised a cutting system using the knives shown in fig 1. A Perspex block containing two parallel blades 4 mm apart (fig 1A,B) is first used to make cuts that define a 4 mm length of biopsy (fig 2B–E). Next a scalpel is used to cut away excess wax (fig 2F). A second cutting tool with blades 2 mm apart (fig 1C,D) is used to make a second cut parallel to the biopsy along the positions of the remaining two black lines (fig 2G,H). The resulting wax block is turned on its side and a final cut with the same knife results in the 4 mm×2 mm×2 mm checker (fig 2I–L). Sides of the checker are marked with red and blue as illustrated during the cutting process to keep track of the position of the face of the checker with the attached biopsy (fig 2G,K).

### Construction of biopsy tissue microarrays

**Figure 4:**
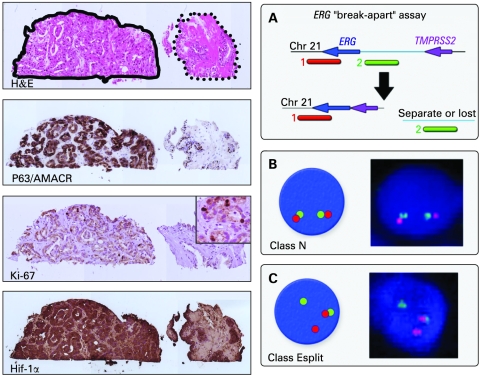
Analysis of sections obtained from a biopsy tissue microarray (TMA). Left panel: Serial sections (×20) from a single biopsy specimen that contained both cancer (surrounded by thick line) and non-neoplastic epithelium (surrounded by dashed line) stained by H&E, p63/AMACR Ki-67 and Hif-1α. The insert shows a magnification of the Ki67 staining. Right panel: FISH detection of *ERG* gene breakpoints. (A) Principle of detection of *ERG* gene status. Interphase nuclei are hybridised to probes that detect sequences 5′ to the *ERG* gene (green) and 3′ to the *ERG* gene (red). (B, C) Results from biopsy TMA slices. The red and green co-localise for normal *ERG* loci (B) and are separated (C) when an *ERG* gene rearrangement occurs.

The uniformity in size of the checkers produced by this procedure enabled us to increase the density of biopsy specimens in each TMA. In one method a wax receptacle block was constructed that contained 54 individual 2 mm×2 mm wells (fig 3A); a single checker is placed in each well. In a second method the wax template contains three rectangular wells of 6 mm×20 mm, 8 mm×20 mm and 10 mm×20 mm that together accommodate 72 checkers (fig 3E). The rubber moulds used for constructing these two templates are shown in fig 3B and 3F, respectively, and the detailed methods for constructing the template are shown in supplementary figs 1 and 2. Following packing of the checkers into the template, warming fuses the wax of the template and checkers into a single block. Examples of biopsy TMAs constructed using these two methods are show in fig 3C and 3G, and the corresponding H&E stained sections are shown in fig 3D and 3H. To ensure that each TMA exhibited a unique pattern of cores, up to 14 blank wax checkers (a blank wax checker lacks an attached biopsy core) were included in each TMA (indicated by a black cross in fig 3). An example of a malignant core from these TMAs sliced and stained with H&E, p63/AMACR (p63/alpha-methylacyl-CoA racemase), Ki-67 and Hif-1α (hypoxia-inducible factor 1, alpha subunit) are shown in fig 4. Around 60% of prostate cancers are reported to contain fusions of the androgen regulated gene *TMPRSS2* to *ERG*^9^ ^11^^–^14 which causes high level expression of 3′-*ERG* gene sequences. In previous studies we have shown that a FISH-based break-apart assay may be used to detect *ERG* gene status in slices taken from conventional TMAs.^9^ ^10^ The same assay can be used to detect *ERG*-gene rearrangements in slices of biopsy TMAs (fig 4A–C).

### Efficiency of analyses using biopsy TMAs

In re-analyses of our previous datasets^7^ collected using biopsy TMAs produced using the original construction technique, we found that of 102 active surveillance patients, 62 yielded cancer that was visible in slices of the biopsy TMA (efficiency = 61%). To assess cancer we examined H&E stained slices 1, 10 and 20 taken from the biopsy TMA: cancer must be present in one or more of these three slices. This low success rate is accounted for because 38 patients had low volume cancer (less than 10% of cancer glands in the original biopsy core). The comparable success rate for biopsies from our previously published study in the MRC RT01 radiotherapy trial,^6^ again using biopsy TMAs produced using our original construction procedure, was 79%. Using the new construction procedure described here the detection rate for cancer in biopsies taken from active surveillance patients was 66%. There were no discernable differences in immunohistochemical results obtained using the old and new biopsy TMA construction techniques.

## DISCUSSION

Over 60 biomarkers have been linked to poorer clinical outcome of human prostate cancer.^15^ However with the exception of serum PSA, none are routinely used in the risk classification of this disease. This is in part a reflection of the inability to rapidly test biomarkers in the precise setting in which they would be used clinically. We have developed a procedure for producing TMAs from prostate cancer needle biopsies taken at the time of diagnosis.^5^ Analysis using this technique allows biomarkers to be examined in clinical specimens taken at the time of diagnosis in patients entered into clinical trials. Indeed we have now confirmed the utility of this approach through analysis of biomarkers in active surveillance trials^7^ and in radiotherapy trials.^6^ However our original approach^5^ was limited because only 20 biopsy cores could be assembled in a single biopsy TMA. In this study we show that through a combination of the development of methods for cutting consistently shaped checkers, and the use of wax templates we can increase this density up to 72 biopsies in each biopsy TMA. This three and a half-fold increase in density reduces the number of TMA slices that need to be produced and examined, and the application of this approach to patients in active surveillance trials has allowed us to construct five biopsy TMAs that contained 287 cores from 108 patients. The construction of TMAs from prostate needle biopsy specimens also allows the multiplex analysis of biomarkers. This is critical because it is likely to be the analysis of a combination of markers rather than of a single marker that will provide the best prognostic information.^16^ In this respect in the context of a radiotherapy trial,^6^ we initially tested three markers (Hif-1α, VEGF and osteopontin) in 201 patients, and the analysis of additional markers is in progress. In conclusion, the methods presented here provided a cheap and simple procedure for constructing TMAs from prostate needle biopsy specimens that allows multiplex analysis of biomarkers in the context of clinical trials.

**Take-home messages**

- There is an urgent need to identify new biomarkers that will aid in improved targeting of radical treatments in patients with prostate cancer.
- To identify such biomarkers it is essential to perform tests on samples taken from the patient at the time of diagnosis, which usually only include blood, urine and trans-ultrasound guided prostate needle biopsy samples.
- A novel method is presented that allows the rapid and uniform production of tissue microarrays from needle biopsies taken from the prostate at the time of cancer diagnosis.
- The biopsy TMAs produced can be used for the multiplex analysis of potential biomarkers detected by immunohistochemistry or by fluorescence in situ hybridisation in prostate needle biopsies taken from patients entered into clinical trials.
